# Seropositivity of Lyme Borreliosis and Associated Risk Factors: A Population-Based Study in Children and Adolescents in Germany (KiGGS)

**DOI:** 10.1371/journal.pone.0041321

**Published:** 2012-08-15

**Authors:** Manuel Dehnert, Volker Fingerle, Christiane Klier, Thomas Talaska, Martin Schlaud, Gérard Krause, Hendrik Wilking, Gabriele Poggensee

**Affiliations:** 1 Department for Infectious Disease Epidemiology, Robert Koch-Institute, Berlin, Germany; 2 National Reference Centre for Borrelia, Bavarian Food and Health Safety Authority, Oberschleißheim, Germany; 3 Institute for Tickborne Diseases, Brieskow-Finkenheerd, Germany; 4 Department of Epidemiology and Health Reporting, Robert Koch-Institute, Berlin, Germany; The Australian National University, Australia

## Abstract

**Background:**

Lyme borreliosis (LB) caused by *spirochetes* of the *Borrelia burgdorferi* sensu lato complex is the most common tick-borne disease in the northern hemisphere. Data on the distribution and on risk factors in Germany are sketchy.

**Methodology/Principal Findings:**

Blood samples of a nationwide population-based cross-sectional study from 2003–2006 in children and adolescents aged 1 to 17 years in Germany (KiGGS) were analysed (n = 12,614) to assess the seroprevalence of anti-*Borrelia* antibodies. Data from standardized interviews were used to assess potential risk factors. First, sera were screened for anti-*Borrelia* antibodies by ELISA. The overall prevalence was 4.8% (95% confidence interval (CI) 4.3–5.4%). Positive and borderline ELISA test results were confirmed by a line blot revealing a combined prevalence of 4.0% (95% CI 3.6–4.5%). Seroprevalence of ELISA was significantly higher in males (odds ratio (OR) = 1.37; CI 1.15–1.63) and in the southern part of Germany (OR = 1.41; CI 1.09–1.83), but significantly lower in children and adolescents with migration background (OR = 0.33; CI 0.24–0.44). Study participants from households with cats had a higher chance of seropositivity (OR = 6.7; CI 5.6–8.0). In a multivariable model the odds of seropositivity increases by 11% for every year of age for boys and 6% for girls.

**Conclusions/Significance:**

This survey is the first nationwide, representative seroprevalence survey of LB in children and young adolescents. The study shows that infections with *Borrelia burgdorferi* are endemic in all parts of Germany despite regional differences. Even at a young age children are exposed to tick bites including seropositivity. Encouraging a thorough check for ticks and promptly removal of ticks are the key public health strategies to reduce the risk of LB and other tick-borne diseases in children and adolescents. Further epidemiological studies are warranted to better understand the burden of disease related to LB.

## Introduction

Lyme borreliosis (LB) is the most prevalent tick-borne zoonosis in the northern hemisphere. It is caused by spirochetes belonging to the *Borrelia* (*B.*) *burgdorferi* sensu lato (s.l.) complex which are transmitted by ticks, in Europe by *Ixodes (I.) ricinus* and, at the eastern range, *I. persulcatus* [1]. Five human-pathogenic genospecies have been described in Europe: *B. burgdorferi sensu stricto*, *B. afzelii, B. garinii, B. bavariensis and B. spielmanii* [2]. The main clinical manifestations of LB include early localized (erythema migrans, borrelial lymphocytoma), early disseminated (multiple erythema migrans, early neuroborreliosis, acute arthritis and carditis) and late disease (acrodermatitis chronica atrophicans, Lyme arthritis and late neuroborreliosis) [3].

Data on the epidemiological situation of Lyme borreliosis in Europe is sketchy. Furthermore, surveillance data are not easily comparable due to different systems used (e.g., voluntary versus mandatory reporting; different reportable disease manifestations, geographic coverage) [4]. In eastern Germany (Brandenburg, Berlin, Mecklenburg-West Pomerania, Saxony, Saxony-Anhalt and Thuringia), erythema migrans, early neuroborreliosis and acute Lyme arthritis are notifiable clinical manifestations. In 2009, the overall annual incidence in these Federal States was 34.7 cases per 100,000 inhabitants. Results from two population-based prospective surveys carried out in 1992 and 1999 the southern part of Germany revealed annual incidences between 111 cases to 260 cases per 100,000 inhabitants [5], [6].

Lyme disease shows a bi-modal age distribution in several European countries, the most affected age groups are children (5 to 9 years) and older citizens (60 to 64 years) [7]–[10]. It is conceivable, that the daily life and play routines of children make them more prone to tick bites. In a prospective study in both conventional kindergartens as well as outdoor kindergartens, so-called “forest kindergartens” in southern Germany, children were followed for up to one year. At least one tick bite was reported by 27% of children attending conventional and 73% for those attending forest kindergartens [11]. According to results from two regional studies in Germany, it is estimated that 4.0 to 5.6% of individuals sero-convert after a tick bite and from this 0.3 to 1.4% develop clinical manifestations [12], [13]. Regional limitation and poor comparability calls for the acquisition of nationwide data on the distribution of *Borrelia* infections in Germany.

Our objectives were to conduct a representative nationwide seroepidemiological survey among children and young adolescents in Germany to assess the seroprevalence of Lyme borreliosis in different population groups and to identify potential risk factors for seropositivity.

## Results

### Study group

The study group consisted of 12,614 children and adolescents, representing 72% of the original study group of KiGGS and 88% (12,614/14,387) of the participants for whom blood samples were available. The unweighted mean age was 10.5 years (range 1–17 years) and 51.3% were male. A total of 35 children (range 6–17 years) reported to have had the diagnosis of Lyme disease.

### ELISA seropositivity

**Figure 1:**
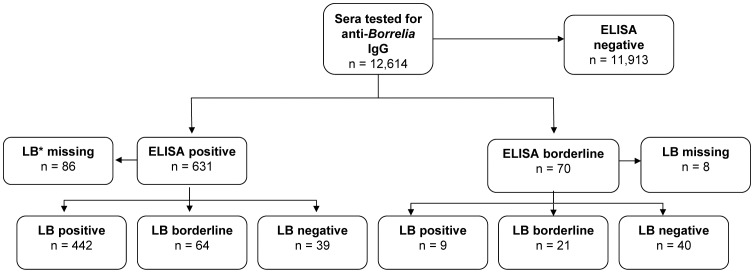
Categorisation of samples tested for anti-*Borrelia* IgG according to the ELISA and line blot test. *LB = Lyme borreliosis.

Out of 12,614 sera, 631 tested positive and 70 borderline by ELISA for IgG against *B. burgdorferi* antibodies (Figure 1). The overall seroprevalence revealed by ELISA was 4.8% (95% CI 4.3–5.4%). Table 1 shows the ELISA seroprevalence stratified for sex, geographical area, age group, migration background, residential area, and presence of pets in a household. A significant higher prevalence was observed in males compared to females (5.5% versus 4.1%). The only significant difference in prevalence between geographical areas was found between the middle and the southern part of Germany with 4.2% and 5.8%, respectively. The seroprevalence increased with increasing age from 1.3% in the age group 1–2 years to 7.1% in the age group 14–17 years. Seroprevalence was significantly lower in children with migration background compared to those without (1.9% versus 5.5%). Study participants with any pets in the household had a significant higher seroprevalence compared to those without pets (5.5% versus 4.2%). A stratified analysis revealed that seroprevalence was particularly high in households with cats compared to those without cats (6.7% versus 4.4%). For dogs and other pets no differences in seroprevalence could be detected.

**Table 1:** Stratified seroprevalence of IgG antibodies against *B. burgdorferi* detected by ELISA in children and adolescents aged 1 to 17 years and results of weighted bivariate logistic regression analysis of potential risk factors for seropositivity, 2003–2006, Germany.

	n* (pos)	Prevalence	95% CI	OR	95% CI	p-Value
**Sex**						
Female (n = 6,147)	274	4.1	3.6–4.8	1		
Male (n = 6,467)	357	5.5	4.8–6.3	1.37	1.15–1.63	0.001
**Geographical area**						
West (n = 8,312)	405	4.7	4.1–5.4	1		
East (n = 4,302)	226	5.6	4.7–6.6	1.20	0.96–1.50	0.100
North (n = 3,317)	146	4.4	3.6–5.5	1.06	0.80–1.39	0.689
Middle (n = 5,557)	257	4.2	3.6–4.9	1		
South (n = 3,740)	228	5.8	4.8–7.0	1.41	1.09–1.83	0.009
**Age group** (years)						
1–2 (n = 898)	13	1.3	0.74–2.3	0.44	0.24–0.81	0.009
3–6 (n = 2,379)	80	2.9	2.3–3.8	1		
7–10 (n = 3,059)	150	5.1	4.2–6.1	1.75	1.28–2.40	0.001
11–13 (n = 2,825)	140	4.6	3.7–5.6	1.57	1.11–2.22	0.010
14–17 (n = 3,453)	248	7.1	6.2–8.2	2.52	1.88–3.38	<0.001
**Migration background**						
No (n = 10,622)	588	5.5	4.9–6.1	1		
Yes (n = 1,953)	40	1.9	1.4–2.6	0.33	0.24–0.44	<0.001
**Residential area**						
Rural area (n = 2,782)	184	7.1	5.6–8.9	1.96	1.44–2.67	<0.001
Small town (n = 3,348)	182	5.4	4.5–6.4	1.47	1.13–1.90	0.004
Mid-sized town (n = 3,683)	156	3.9	3.1–5.0	1.05	0.77–1.43	0.758
Metropolitan (n = 2,801)	109	3.7	3.1–4.5	1		
**Pet in household**						
No pet (n = 6,374)	283	4.2	3.6–4.9	1		
Any pet (n = 5,982)	336	5.5	4.8–6.3	1.34	1.10–1.62	0.003
**Dog**						
No (n = 10,346)	510	4.7	4.2–5.3	1		
Yes (n = 1,978)	106	5.4	4.4–6.6	1.15	0.92–1.43	0.225
**Cat**						
No (n = 9,963)	460	4.4	3.9–5.0	1		
Yes (n = 2,361)	156	6.7	5.6–8.0	1.56	1.25–1.94	<0.001
**Other animals**						
No (n = 10,224)	503	4.7	4.2–5.3	1		
Yes (n = 2,132)	116	5.4	4.3–6.6	1.14	0.90–1.44	0.272

* unweighted.

### Combined ELISA and line blot seropositivity

Applying the rules for combining results (as described in methods) the overall seroprevalence was 4.0% (95% CI 3.6–4.5%). Table 2 shows the combined ELISA and line blot seroprevalence stratified for sex, geographical area, age group, migration background, residential area, and presence of pets in a household. The results are qualitatively the same as for the ELISA results.

**Table 2:** Combined ELISA and line blot test results: Stratified seroprevalence of IgG antibodies against *B. burgdorferi* in children and adolescents aged 1 to 17 years and results of weighted bivariate logistic regression analysis of potential risk factors for seropositivity, 2003–2006, Germany.

	n* (pos)	Prevalence	CI 95%	OR	CI 95%	p-Value
**Sex**						
Female (n = 6,101)	215	3.3	2.8–3.8	1		
Male (n = 6,419)	300	4.7	4.1–5.4	1.48	1.22–1.80	<0.001
**Geographical area**						
West (n = 8,248)	334	3.9	3.4–4.5	1		
East (n = 4,272)	181	4.5	3.7–5.5	1.17	0.91–1.49	0.219
North (n = 3,294)	119	3.6	2.8–4.5	1.03	0.76–1.37	0.866
Central (n = 5,522)	206	3.5	2.9–4.1	1		
South (n = 3,704)	190	4.9	4.1–5.9	1.44	1.10–1.88	0.007
**Age group** (years)						
1–2 (n = 893)	4	0.4	0.13–1.2	0.17	0.06–0.51	0.002
3–6 (n = 2,364)	61	2.3	1.7–3.2	1		
7–10 (n = 3,033)	119	4.1	3.3–5.0	1.79	1.24–2.59	0.002
11–13 (n = 2,809)	119	4.0	3.2–4.9	1.74	1.18–2.55	0.005
14–17 (n = 3,421)	212	6.2	5.3–7.1	2.77	1.93–3.98	<0.001
**Migration background**						
No (n = 10,622)	486	4.6	4.1–5.2	1		
Yes (n = 1,953)	26	1.3	0.84–1.9	0.26	0.18–0.39	<0.001
**Residential area**						
Rural area (n = 2,745)	141	5.7	4.6–7.0	1.98	1.45–2.71	<0.001
Small town (n = 3,322)	153	4.5	3.7–5.5	1.57	1.16–2.11	0.003
Mid-sized town (n = 3,666)	136	3.5	2.7–4.5	1.19	0.84–1.68	0.322
Metropolitan (n = 2,787)	85	2.9	2.3–3.7	1		
**Pet in household**						
No pet (n = 6,323)	216	3.3	2.8–3.9	1		
Any pet (n = 5,940)	289	4.8	4.2–5.5	1.46	1.19–1.80	<0.001
**Dog**						
No (n = 10,268)	412	3.9	3.4–4.4	1		
Yes (n = 1,964)	91	4.8	3.9–5.9	1.25	0.99–1.59	0.062
**Cat**						
No (n = 9,885)	361	3.5	3.1–4.0	1		
Yes (n = 2,347)	142	6.2	5.2–7.4	1.56	1.25–1.94	<0.001
**Other animals**						
No (n = 10,149)	412	4.0	3.5–4.5	1		
Yes (n = 2,114)	93	4.2	3.3–5.2	1.05	0.81–1.35	0.709

* unweighted.

#### Univariable analysis

In the univariable logistic regression analysis for ELISA seropositive cases, sex, geographical area, age group, migration background, residential area, and the presence of pets in general and in particular cats in household were potential risk factors for seropositivity (p<0.25). As a proxy for behavioural factors, outdoor activities were analysed in a subgroup analysis (children of 3–10 years of age, data not shown). The analysis did not reveal a significant association between frequency of outdoor activities and seropositivity.

#### Adjusted Analyses

Results of the multivariable analysis of ELISA seropositive cases are reported in Table 3. The model contains a significant interaction between sex and age as continuous variable. The odds of seropositivity increases by 11% for every year of age for boys and 6% for girls. At the mean age of 10.5 years the odds for seropositivity is 27% higher in boys compared to girls. The model shows that in comparision to the middle part of Germany, children in the southern part had a 30% percent higher odds to be seropositive. The odds of being seropositive was 65% lower in children with migration background. Study participants living in rural areas or small towns had a 29% higher odds to be seropositive. Study participants having a cat in the household had a 30% higher odds to be seropositive, whereas the presence of dogs or other pets was not related to an increased chance for seropositivity.

**Table 3:** Results of weighted multivariable logistic regression analysis of potential risk factors for ELISA and combined ELISA and line blot seropositivity (n = 12,297 after exclusion of participants with missing data).

	ELISA			Combined ELISA and line blot results		
	OR	CI 95%	p-value	OR	CI 95%	p-value
**Sex**						
Female*	1			1		
Male*	1.27	1.06–1.53	0.010	1.35	1.10–1.66	0.004
**Age** (years) (Interaction with sex)						
Female	1.06	1.03–1.09	<0.001	1.07	1.03–1.11	<0.001
Male	1.11	1.08–1.14	<0.001	1.13	1.09–1.16	<0.001
**Migration background**						
No	1			1		
Yes	0.35	0.25–0.47	<0.001	0.28	0.19–0.42	<0.001
**Residential area**						
Rural area/small town	1.30	1.03–1.63	0.026	1.2	0.95–1.52	0.129
Mid-sized town/Metropolitan	1			1		
**Geographical area**						
North	1.03	0.79–1.36	0.814	0.99	0.74–1.33	0.949
Central	1			1		
South	1.30	1.01–1.67	0.044	1.34	1.03–1.75	0.028
**Cat**						
No	1			1		
Yes	1.30	1.04–1.63	0.024	1.50	1.19–1.90	0.001

* at mean age of 10.5 years.

Being male, higher age, residence in rural area/small town, residence in southern part of Germany and the presence of a cat in the household were significantly associated with an increased chance for seropositivity.

The results of the multivariable analysis of risk factors based on the combined ELISA and line blot results (Table 3) did not differ from the multivariable analysis based on the ELISA only with regard to sex, migration background, residence in southern Germany and the presence of cats in the household. The presence of dogs in the household increased the chance to be seropositive, however, the result was not statistically significant.

## Discussion

We describe the results of the first nationwide, representative serosurvey on Lyme borreliosis in children and adolescents. Lyme borreliosis is prevalent in all regions of Germany and seropositive children can even be found in the youngest age groups. Children and young adolescents living in rural areas or in small-sized towns were at higher risk of having contracted an infection with *Borrelia burgdorferi* s.l. Furthermore, residence in the southern part of Germany and being male increased the risk of seropositivity. On the other hand, having a migration background reduces the chance for seropositivity.

Recent seroprevalence studies providing ELISA and immunoblot results showed a considerably lower proportion of confirmation compared to our study (81% confirmation of ELISA results by immunoblot). A serosurvey amongst United States military personnel identified 16.5% positive samples by ELISA (1,594/9,673 samples), but only 0.12% could be confirmed by Western blot [14]. The seroprevalence study among adult forestry workers and farmers in Turkey showed an ELISA seropositivity of 10.9% and a Westernblot positivity of 1.1% [15].

Screening of IgG antibodies against *B. burgdorferi* in blood donors as a proxy for the presence in the healthy population showed seroprevalences of 2.7% both in Hamburg and Bavaria [16], [17]. In France (3.2%) [18], Italy (4.9%) [19] and Romania (4.3%) [20], similar proportions of seropositive individuals among blood donors were assessed. In population-based surveys, higher seroprevalences were seen in Germany (Berlin: 8%, n = 3,736 [21]; Bavaria: 15%, n = 4,896 [22]; Baden-Württemberg: 16.9%, n = 1,228 [5]) and Finland (19.3%, n = 3,248 [23]). In individuals with higher risk of exposure to ticks such as forestry and agricultural workers seroprevalences between 8% and 52% have been described [15], [18], [19], [24]–[26]. In a cross-sectional study in northern Sardinia 14-year-old teenagers were screened for IgG and IgM antibodies by ELISA test against *B. burgdorferi*. The seroprevalence detected was 6.1% (n = 443 [27]). A population-based study in Southeast Sweden showed a *Borrelia* IgG antibody seroprovalence assessed by ELISA of 3.2% (n = 2,000) in five-year old Swedish children [28]. These data are within the range of 2.9% (CI 2.3–3.8) observed in the KiGGS age group (3–6 years) (Table 1). In Lower Saxony in a regionally representative study a seroprevalence of 2.6% (n = 574) was determined in children aged 0 to 13 years [29]. Variations between studies may be ascribed to different test systems applied and the age-range of the study population.

Besides these studies, children and young adolescents were either underrepresented or not included in seroprevalence surveys, or the data were not presented according to age.

In Germany the knowledge of the epidemiological situation of Lyme borreliosis is incomplete. Routine surveillance data are available for the eastern part of Germany. The results of our study show that Lyme borreliosis is endemic throughout Germany, furthermore, children and adolescents living in the southern part of Germany have a higher chance to be infected. The incidence rates in Southern Germany are likely to be higher compared to surveillance area in East Germany.

In children aged 1 year and older the risk of seropositivity increased by 6–11% each year and at the age of 17 years, 7% of children and young adolescents have already experienced at least one tick bite with successful seroconversion. However, it has to be kept in mind that IgG antibodies can persist for over 10 years [30]. Therefore the true age of a child or young adolescent at the time of infection cannot be determined and the seroprevalences seen in the different ages reflect the cumulative incidence proportion. Children and young adults with migration background were less likely to be seropositive. One possible explanation for this finding could be less exposure to ticks due to different factors such as origin from non-endemic country or behavioural factors.

In our study, residence in rural areas and small towns was a risk factor for seropositivity. Residence in forested areas has been identified as risk factor for Lyme borreliosis in the USA and in Europe [31]–[33]. Our findings are in concordance with the results of population-based studies from the southern part of Germany revealing high incidences of Lyme borreliosis in rural areas of Baden-Württemberg and Bavaria [6], [12]. However, in our study we observed seroprevalence as high as 3.7% in metropolitan areas. Foci of borrelia-infected ticks have been demonstrated in urban parks and private gardens in Europe with infection rates up to 55% [34]–[38]. Furthermore, rodents such as the Norway rat (*rattus norvegicus*) and the black rat (*rattus rattus*) which can be a pest in metropolitan cities can act as reservoir host possibly enhancing the risk of Lyme borreliosis [39].

The association found in the bivariate and multivariable analysis between the presence of cats in the household and seropositivity was unexpected. Although households in rural areas were more likely to keep cats, the association remained in the multivariable analysis. An association between seropositivity and pet-owning could not be shown in Italian teenagers; however the study does not provide data on the type of pets kept in a household [27].

It has been shown that infection with *B. burgdorferi* occurs in highly focussed areas [7], [8]. In Germany, within individual states, a distinct heterogeneity of incident cases can be seen in the counties of a single state [40]. It can be assumed that the risk of infection is not uniformly distributed on a small area level but depends e.g. on the suitability of habitats for ticks. Plausible risk factors such as outdoor activities and ownership of dogs as a proxy for possible exposure during walks were in our analysis on national level not associated with Lyme borreliosis. Cats are underestimated as risk factors. It can be hypothesized that cats act as optional intermediate vectors infestated by ticks during the day and transferring them to the keeper while stroking and cuddling.

### Limitations

As the KiGGS study recruited only infants and children we have no data on the adult population. This deficiency should be approached in the future. Due to the study design - including different cluster centre - we were not able to identify spatial small-scale variations of infectious risk in Germany. Thus this study cannot replace detailed ecological studies providing geographical information on the occurrence of likely tick exposure and the prevalence of Bb sl in ticks. These data could be further used to identify spatial patterns of areas with increased risk of contracting infection with Bb sl. Still, our results are valid on a large scale regional level which already revealed geographical differences.

Seroconversion is not equivalent with clinical manifestation of disease and it has to be assumed that inapparant infections without significant symptoms and reliable clinical diagnostic are unequal distributed among the groups investigated in this study. Thus, the differences in seroprevalence between groups are maybe not reflected by differences in the real disease burden between groups [41]. The positive and negative predictive values of the ELISA could have had a significant influence on the prevalence estimates. However, the agreement between the two independent test systems applied in our study is high. This indicates that there are no weaknesses in one of the both test. Additionally, a significant proportion of false-positives would have been also been observed in the youngest age cohort. But the prevalence is lowest in the youngest age group (Table 1) and even 0.5% in the one-year-old which reflects an approximate zero line as a baseline for cumulative incidence proportion in older age cohorts. Further studies have to be initiated to fully understand the disease burden of Lyme borreliosis in Germany.

### Conclusions

Lyme borreliosis is endemic in all regions of Germany and even at a young age, children are exposed to tick bites resulting in an infection with *Borrelia burgdorferi*. In areas with high incidences public health interventions such as information campaigns targeted at parents and children should be carried out to provide information about potential risk factors as well as preventive measures.

## Materials and Methods

### Ethics Statement

Participants above 14 years of age and all parents provided written informed consent prior to the taking of blood samples and the interview. This study was approved by the Ethical Clearance Committee of the Medical school Charité, Humboldt-University, Berlin, Germany and by the Federal Office for Data Protection, Germany.

### Study Group

The German Health Interview and Examination Survey for Children and Adolescents, KiGGS, was conducted between 2003 and 2006 to collect comprehensive data on the health status of children and adolescents aged 1 to 17 years with principle residence in Germany [42]. Participants were enrolled in two steps: first, 167 study locations (sample points) were chosen; second, subjects were randomly selected from the official registers of local residents. A total of 17,641 children and adolescents were surveyed, 8,985 boys and 8,656 girls (response rate 66.6%). Analysis of non-responder questionnaires revealed that the collected data provided comprehensive and nationally representative evidence on the health status of children and adolescents. In order to confirm that estimates derived from the KiGGS study were representative at the national level, survey weights were calculated to adjust for deviations between the design-weighted net sample and German population statistics based on cross-classifications by age, sex, residence in western or eastern Germany, and nationality (German vs. non-German). Weighting mainly resulted in correction for differences in age structure and disproportionately higher sample size in eastern versus western Germany. A detailed description of the survey design and weights has been given elsewhere [42]. In this study the following independent variables were included from the data generated by interviews with the parents and/or children/adolescents: sex, age, residence, outdoor activities (frequency of playing outside, sport activities), presence of pets in the household, and migration status.

### Serological assays

The sera were tested at the National Reference Centre for Borrelia for the presence of anti-Borrelia IgG antibodies.

#### ELISA

For screening an enzyme-linked immunosorbent assay (ELISA) (Enzygnost Lyme link VlsE/IgG, Siemens Healthcare Diagnostics GmbH, Eschborn, Germany) was used. This quantitative ELISA is based on a detergent extract from cultured *B. afzelii* (strain PKo) mixed with recombinant VlsE from *B. burgdorferi s.s.* (strain B31), *B. afzelii* (strain PKo), and *B. bavariensis* (strain PBi). The test was automatically processed on a BEP®III (Siemens Health Diagnostics GmbH, Eschborn, Germany) and interpreted as recommended by the manufacturer. Validation studies for this ELISA have been published [43], [44].

#### Line blot

As a confirmatory assay a line blot was performed (Borrelia Europe plus TpN17 LINE, IgG, Virotech, Rüsselsheim, Germany). This test includes the purified antigens OspC, DbpA, and p83 (all from *B. afzelii* strain PKo) and the recombinant antigens VlsE (from *B. burgdorferi* s.s. strain B31 and *B. garinii* strain IP90), BmpA (PKo), DbpA (from *B. garinii* strain PBr, *B. bavariensis* strain PBi, and *B. spielmanii*). Antigens are bound separately to a nitrocellulose membrane either as single antigens or in case of VlsE and DbpA as a mix of the respective antigens. The test was performed and interpreted according to manufacturers recommendations.

### Seropositivity

Results of ELISA and line blot were categorised as negative, borderline or positive. A subset of samples - determined by the availability of sera - with a positive or borderline ELISA test result, was subjected to line blot to confirm the test result. Results of ELISA as well as combined results of ELISA and immunoblot were considered. To combine results the following rules were applied: In case of both a positive ELISA and immunoblot test result, the sample was categorised as positive. In case of a borderline test results from both tests, the sample was categorised as negative. In case of discordant test results, the sample was categorised as negative, except in cases involving a borderline ELISA test result and a positive immunoblot result or vice versa; then the sample was categorised as positive. Samples with borderline or positive result in ELISA, that due to lack of sera, missed out on immunoblot results were categorised as missing. Samples with negative test results in ELISA, that were not further tested by immunoblot, were categorised to be negative.

### Statistical analysis

All statistical analyses used sampling weights and accounted for the cluster structure of the multi-stage survey design. We estimated point prevalences and corresponding 95% confidence intervals (CI). Differences in prevalences are assessed by the Wald test (univariable logistic regression) which was applied to identify potential risk factors for seropositivity. Predictors with p<0.25 were considered for multivariable analysis. Stepwise multivariable logistic regression was used to investigate independent risk factors for seropositivity. Results are presented as odds ratios with 95% confidence interval. All possible twoway interaction terms were tested separately. Reported p-values are two sided and p<0.05 was considered statistically significant. Statistical analyses were performed with Stata 10.1 (StataCorp LP, TX, USA).

### Categorization for analysis purposes

Analyses were based on the following definitions of seropositivity: i. ELISA seropositivity: samples with a positive ELISA test result were regarded as seropositive; ii. combined ELISA and line blot test seropositivity: applying the rules described above samples were categorized as seropositive. Age groups were defined as 1–2, 3–6, 7–10, 11–13 and 14–17 years of age. For the geographical analysis two different approaches were used (the names of Federal States are given in parenthesis): i. Categorising Germany into an eastern (Berlin, Brandenburg, Mecklenburg-West Pomerania, Saxony, Saxony-Anhalt, Thuringia) and western (Baden-Württemberg, Bavaria, Bremen, Hamburg, Hesse, Lower Saxony, Northrhine-Westfalia, Rhineland-Palatinate, Saarland, Schleswig-Holstein) part; ii: Categorising Germany into a northern (Schleswig-Holstein, Hamburg, Lower Saxony, Bremen, Berlin, Brandenburg, Mecklenburg-West Pomerania), middle (Nordrhine-Westfalia, Hesse, Saxony, Saxony-Anhalt, Thuringia) and southern (Rhineland-Palatinate, Baden-Württemberg, Bavaria, Saarland) part. The definition for residential areas is “rural area” (<5,000 inhabitants), “small town” (5,000 to <20,000 inhabitants), “mid-sized town” (20,000 to <100,000), and “metropolitan” (>100,000 inhabitants), respectively. For multivariable analysis, residential areas were regrouped, merging the categories “rural area” and “small town” as well as “mid-sized town” and “metropolitan”. Study participants in KiGGS were classified as migrants if one of the following criteria was met: study participant migrated to Germany and at least one parent was born outside Germany; or both parents migrated to Germany or neither parent has German citizenship [45]. In this study participants with migrant status or having no German citizenship were classified as having a migration background.
